# Comparing the health of non-binary and binary transgender adults in a statewide non-probability sample

**DOI:** 10.1371/journal.pone.0221583

**Published:** 2019-08-27

**Authors:** Sari L. Reisner, Jaclyn M. W. Hughto

**Affiliations:** 1 Division of General Pediatrics, Boston Children’s Hospital, Boston, Massachusetts, United States of America; 2 Pediatrics, Harvard Medical School, Boston, Massachusetts, United States of America; 3 Department of Epidemiology, Harvard T.H. Chan School of Public Health, Boston, Massachusetts, United States of America; 4 The Fenway Institute, Fenway Health, Boston, Massachusetts, United States of America; 5 Departments of Behavioral and Social Sciences and Epidemiology, Brown University School of Public Health, Providence, Rhode Island, United States of America; 6 Center for Health Equity Research, Brown University, Providence, Rhode Island, United States of America; University of California Los Angeles, UNITED STATES

## Abstract

**Background:**

In the U.S., non-binary refers to transgender people who have a gender identity not aligned with their assigned sex at birth, and who identify outside of the traditional male-female binary, such as genderqueer, genderfluid, or gender nonconforming. Few data are available to characterize the health of non-binary adults.

**Methods:**

The current study sought to fill this gap by conducting a secondary analysis of data from a non-probability sample of transgender and/or gender nonconforming adults in Massachusetts (sample mean age 32.6 years, 63% female assigned sex at birth; 79.4% white non-Hispanic/Latinx). Multivariable models were fit to compare non-binary (e.g., genderqueer) vs. binary (e.g., man/trans man, woman/trans woman) respondents across a range of social and health indicators.

**Results:**

Overall, 40.9% identified their gender identity as non-binary. Non-binary respondents significantly differed from binary respondents on (all *p*<0.05): demographics (younger age, more female assigned sex at birth); gender affirmation (older age of identity recognition, lower current uptake of and future desires for medical gender affirmation); healthcare utilization (lower rates of being up-to-date in annual wellness visit, less mental healthcare utilization in past year); mental health and substance use (higher past-week depressive distress, higher hazardous alcohol use); social history (more unstably housed, more current students), violence victimization (lower rates of lifetime intimate partner violence), and social support (less family support).

**Conclusion:**

Gender diversity, including whether people endorse a binary or non-binary gender identity, is a prevalent and an important aspect of transgender health. Demographic measures of gender identity that include binary and non-binary response options are recommended to inform future research and clinical care.

## Introduction

Transgender people have a current gender identity that differs from the sex assigned to them at birth, and comprise an estimated 1.4 million adults in the U.S.[1] In recent years, transgender public health, clinical epidemiology, and medicine have begun to garner increasing interest, attention, and support as “legitimate” areas of scientific inquiry and domains for clinical care innovation.[2–4] Accompanying this growth, and perhaps bi-directionally influencing it, has been a paradigmatic shift in the field of transgender health moving away from conceptualizing transgender as a “disorder” and toward conceptualizing transgender as an “identity.”[3] Indeed, as gender diversity is becoming increasingly de-pathologized, there is beginning to be greater recognition of the proliferation of gender identities and heterogeneous gender presentations that exist for transgender people.[5, 6] However, very little research and empirical data are available to understand similarities and differences in the health risks, conditions, and needs of diverse groups of transgender people, including those who identify outside the gender binary.[7–10]

*Non-binary* refers to transgender people who have a gender identity that does not align with their sex assigned at birth and who identify outside of the traditional female-male gender binary, such as genderqueer, genderfluid, or gender nonconforming.[8] Non-binary people may have an identity and/or expression that is either feminine or masculine, both feminine and masculine, or neither. Non-binary individuals may also be pangender (two or more genders), bigender (female and male), agender (without any gender), neutrois (neutral or genderless), and many other diverse gender identities. In the 2015 U.S. Transgender Survey (USTS), a U.S. national convenience sample of more than 22,000 transgender and gender nonconforming adults, more than one third (35%) of respondents identified as non-binary.[11] Non-binary-identified transgender people may have different sociodemographic characteristics than binary transgender people (e.g., those who identify with a binary gender such as transgender men or transgender women).[12] Non-binary participants tend to be younger than binary individuals, and more likely to have a non-heterosexual/straight sexual orientation vs. not.[13, 14] Other studies have not found significant differences between binary vs. non-binary gender groups by race, socio-economic status (SES), or other key characteristics known to shape health, such as relationship status, student status, sexual behavior, sex work, housing status, and military service.[11] A recent review of non-binary health research identified fewer than one dozen published studies.[10] Additional research is needed to characterize non-binary and binary people in order to enhance epidemiologic conclusions that can be drawn about these groups.

Minority stress frameworks have been used to understand health-related differences between transgender and cisgender (non-transgender) people,[15–17] wherein poor health is caused by the differential distribution of social stressors (e.g., stigma, mistreatment)[18] resulting from being a member of a marginalized group. However, minority stress frameworks also motivate hypothesized differences in the social history, healthcare utilization behaviors, and health outcomes of non-binary vs binary transgender people.[19] People generally lack basic knowledge and competency of non-binary gender identities and experiences, including family members, employers, healthcare providers, and larger societal systems.[20] Not conforming to the male-female gender binary or gender social norms may expose non-binary transgender people to gender-related stressors from multiple sources. Conversely, it could be that non-binary people have better health because subverting gender norms or not identifying with societal male-female stereotypes offers freedom from gendered expectations and removes some stressors. Therefore, in addition to exploring the socio-demographic differences of transgender people who identify as binary vs. non-binary, understanding differences in healthcare utilization and health by gender identity sub-group is also important for ensuring access to necessary care for transgender people, including medical gender affirmation therapies and other affirming services such as mental health counseling.

While genderqueer and other non-binary transgender individuals have been defined as those who typically do not desire to medically affirm their gender,[21] empirical research suggests that many transgender individuals who identify outside the gender binary may still seek hormones or surgery.[11, 22] Indeed, findings from the USTS show that while 95% of binary transgender participants wanted hormone therapy, 49% of non-binary individuals also desired hormone therapy, although only 13% of these individuals had received it, relative to 71% of binary individuals.[11] These gender identity differences in desire for gender affirmation vs. actual hormone utilization could suggest unique access to care barriers for non-binary transgender individuals, such as stigma in clinical care settings, including the provision of medical gender affirmation in accordance with gender binary protocols.[20, 23] To that end qualitative data reveal that non-binary participants tend to be less likely than binary participants to have discussed their gender identity with their provider or disclose being transgender).[6, 24] In the context of research demonstrating that healthcare avoidance due to past or anticipated discrimination from one’s healthcare provider influences access to care for transgender individuals,[18] experiences of discrimination and avoidance could be driving differences by gender identity in access to gender affirming care. Descriptive data support that possibility, as 39% of binary vs. 24% of non-binary participants in USTS had experienced negative healthcare experiences, although differences in avoidance of care due to discrimination were smaller for non-binary participants (20%) vs. binary participants (27%).[11] Similarly, in another study of 150 transmasculine adults of which 23% were non-binary, 68.7% of the sample reported experiencing mistreatment in healthcare settings in their lifetime which was positively associated with healthcare avoidance in the past 12 months.[25] These findings highlight the importance of using more robust statistical methods to further explore healthcare utilization and the mechanisms driving gender identity differences in barriers to care, including discrimination and other forms of stigma.

Despite that non-binary people encompass a sizable proportion of the transgender population, much of the research characterizing the health and wellbeing of transgender people either treats transgender individuals as a homogenous population, or at best, stratifies transgender individuals by gender spectrum (e.g., female-to-male/transgender men/trans masculine vs. male-to-female/transgender women/trans feminine), particularly in research on discrimination and violence. Indeed, research demonstrates variable rates of violence across transgender communities with some studies showing a similar or higher prevalence of lifetime sexual and physical assault among trans masculine individuals,[26, 27] while other studies demonstrate a higher prevalence of physical violence[28] and sexual assault[29, 30] among trans feminine individuals. Among the little research that does exist by binary vs. non-binary identity, findings are mixed. Some research has found higher prevalence of harassment, trauma, and sexual assault for non-binary young adults relative to those who identify as binary.[19] The USTS survey showed only slight differences in the prevalence of lifetime sexual assault by binary gender identity (44% binary vs. 46% non-binary); however, binary individuals were more likely to be physically attacked (29%) and sexually assaulted (15%) in grades kindergarten through 12^th^ grade, relative to non-binary individuals (16%). In a clinical sample of patients at an urban health center, past-12 month intimate partner violence (IPV) rates were elevated for transgender women (12.1%), transgender men (6.6%), and non-binary individuals (8.2%) relative to cisgender women (2.7%) (p<0.05).[31] Another study of college students accessing college-based mental health counseling from the Center for Collegiate Mental Health’s 2012–2016 database, found non-binary transgender individuals had higher rates of harassment, sexual abuse, and exposure to traumatic events than binary transgender students.[19] Understanding gender differences in experiences of victimization and violence within the transgender community is critical, as these experiences have been linked to poor mental health in transgender populations.[16, 32]

Research shows that transgender individuals relative to cisgender individuals experience heightened levels of depression, anxiety, substance use, suicidality, and poor mental and overall self-rated health.[16, 33–38] The USTS reported that binary participants (49%) were more likely to report current serious psychological distress than binary participants (35%), yet were slightly less likely to report lifetime suicide attempts (43% binary vs. 39% non-binary). [11] While family and peer support have been shown to buffer against the mental health costs of violence and victimization,[39, 40] research suggests that rejection by one’s family and peers is common for transgender individuals. In the USTS sample, 46% of transgender participants reported experiencing some form of family rejection, 77% being bullied for being transgender as a child, and 19% of binary vs. 15% of non-binary individuals having to leave school due to harassment. In a smaller study of 150 trans masculine individuals, relative to those with a binary gender identity, those with a non-binary gender identity had higher odds of both clinically significant depression and anxiety symptoms, though results only approached significance (*p* = 0.06) as the analysis was likely underpowered.[25] Research is needed to examine multiple indicators of support including the degree (continuous instead of categorical measure of support), extent (e.g., number of friends), and timing (current vs. childhood) of support that transgender individuals experience by gender identity.

Lack of research exploring the differences in the health of non-binary transgender people relative to binary transgender people, may obfuscate the unique health needs of this sub-population. Further, the fields of public health and epidemiology need to consider gender diversity beyond the gender binary. In addition to the categories “transgender men” and “transgender women” that are often used in public health, non-binary vs binary represent another gender axis that should be included in research in order to shed light on health disparities faced by transgender people relative to cisgender individuals. In order to fill this research gap, the current study sought to characterize the health and wellbeing of non-binary adults in a statewide sample of transgender respondents in Massachusetts, comparing the demographic characteristics and health of those identifying their gender identity as non-binary with those self-reporting a binary identity. Such data are urgently needed to understand the epidemiology and health needs of this community, guide future public health efforts, and inform responsive clinical care.

Based on the review of research and application of minority stress frameworks (see above), this study hypothesized that non-binary respondents would differ from binary respondents on demographics (younger age, more female assigned sex at birth, more sexual minority identified, higher educational engagement, more privately insured), gender affirmation (lower levels of internalized transphobia, less current medical gender affirmation, higher visual gender nonconformity), healthcare utilization (less routine engagement in healthcare, more teaching of healthcare providers to obtain appropriate care), mental health (more depression and anxiety, more unmet need for mental health services), substance use (greater alcohol misuse), and violence victimization (more experiences of victimization). No significant differences were anticipated in social history or social support for non-binary compared to binary respondents.

## Methods

This is a secondary analysis of data from Project VOICE (Voicing Our Individual and Community Experiences), a stress and health needs assessment of transgender and gender nonconforming adults in Massachusetts, conducted between March and December 2013 by the Fenway Institute at Fenway Health (Fenway) and the Massachusetts Transgender Political Coalition (MTPC). This non-probability sample study used a participatory population perspective,[41] grounded in community-based participatory research principles, to work “with” not “on” transgender communities in the Commonwealth. The community sample was recruited using bi-model methods, either in person (via community events, programming, and gatherings) or online (via electronic listservs, emails, website postings at Fenway and MTPC, and social networking sites). Eligibility criteria were: (1) self-identified as transgender or gender nonconforming; (2) ages 18 years or older; (3) living in Massachusetts (or had lived in Massachusetts for at least 3 months of the past year); (4) had not previously completed the survey; (5) was able to read and understand English or Spanish. Whenever possible, the study utilized validated questions or adapted survey items from earlier research to ensure the comparability of findings, including those from such sources as the U.S. National Transgender Discrimination Survey[42] and Behavioral Risk Factor Surveillance System (BRFSS).[43] Project VOICE was approved by the Fenway Institutional Review Board (IRB). Additional details regarding study methodology are reported elsewhere.[44]

### Measures

#### Independent variable/ exposure: Non-binary vs. binary gender identity

Gender identity was assessed using the recommended two-step method[45] with two items: (1) assigned sex at birth (female, male) and (2) current gender identity (man, female-to-male, trans man, trans male/ woman, male-to-female, trans woman, trans female/ genderqueer, gender variant, gender nonconforming, another gender). Participants were asked to select a single response option that best described their current gender identity. Participants were categorized as having a binary gender identity (e.g., male/trans male, female/trans female) or a non-binary gender identity (genderqueer, gender variant, gender nonconforming) based on their response to the current gender identity item. The two items were also cross-tabulated to categorize participants as trans feminine spectrum (*n* = 167) or trans masculine spectrum (*n* = 285).

#### Dependent variables

Demographic characteristics: Age in years (continuous), race/ ethnicity (white, Black, Latino, Other, Mixed), sexual orientation (sexual minority vs. not), low education (high school diploma vs. college or above), current student status (yes vs. no), low income (<$35K annually, $35K annually or above), health insurance (public, private, other), survey mode (online, in-person).

Gender affirmation characteristics: Age first recognized gender identity (“How old were you when you first became aware that you were transgender and/or gender nonconforming?”). Participants were asked whether a doctor had ever diagnosed them with gender identity disorder or gender dysphoria (yes, no). Social gender transition was assessed with the following item: “Do you consistently present (live ‘full time’) in your identified gender?” (yes, no). Medical gender affirmation was assessed with the following item: “Have you accessed any transgender-related medical interventions to affirm your gender (e.g., hormones, surgeries)?” (1 = yes yes, no) followed by items asking about intervention types (hormones, chest surgery, abdominal surgery, genital surgery, other procedures). Non-prescription hormone use (yes, no), silicone use (yes, no). Visual gender-nonconforming (GNC) expression was assessed with the following item: “People can tell I’m transgender or gender nonconforming even if I don’t tell them.” This item was assessed on a 5-point Likert scale from 0 (never) to 4 (always). The item was coded into low GNC (never or occasionally), moderate GNC (sometimes), and high GNC (most of the time or always).

Healthcare utilization behaviors and experiences: Participants were asked when they had their last annual wellness visit to a doctor (within last year, within the last 1–2 years, within 3–5 years, 5+ years ago). Healthcare experiences in the last 12 months were queried, including: having to teach a doctor about transgender health in order to get appropriate care (yes, no), postponed or did not try to get medical care when needed it resulting in a medical emergency to the emergency room or urgent care clinic to get immediate help (yes, no), postponed healthcare when sick or injured (yes, no), postponed or did not try to get check-ups or other preventive medical care (yes, no), refused treatment or medical care (yes, no), and had one or more experiences of mistreatment or discrimination in healthcare (yes, no).

Social history, violence victimization, and social support: Respondents were asked about whether or not they were sexually active (yes, no), currently partnered (yes, no), had biological and/or adopted children (yes, no), had ever been homeless or unstably housed (yes, no), had ever engaged sex work or transactional sex for money, food, drugs, or other basic needs (yes, no), and had ever served in the military (yes, no). Violence victimization was assessed including: ever intimate partner violence (IPV) (“ever been slapped, punched, kicked, or otherwise physically or sexually hurt by your spouse (or former spouse), a boyfriend/girlfriend, or some other intimate partner”; yes, no); physical or sexual abuse as a child under age 15 (physical and/or sexual), coded as no abuse, yes both physical and sexual abuse, yes physical abuse only, yes sexual abuse only); and bullying in-person in childhood under age 18 (0 = Never to 4 = All the Time). Family support of gender was asked (“In general, how supportive of your gender identity or expression is your family?” 0 = Not at all supportive to 4 = very supportive). Social support was assessed including the number of close friends (people you can confide in) and number of close friends who are transgender.

Mental health: Internalized transphobia was assessed through a single-item question capturing the intersection of alienation and shame[46] (“I wish I was not transgender and/or gender nonconforming”, 1 = strongly disagree to 5 = strongly agree). Participants were asked whether they had ever in their lifetime engaged in self-harm (self-injurious behavior without suicidal intent; yes, no) and whether they had ever attempted suicide (yes, no). The validated 10-item short form of the Center for Epidemiologic Studies Depression Scale (CES-D-10) was used to screen respondents for past-week depression; participants were categorized as meeting current clinically significant depressive distress (yes score 10+, no score <10).[47] Respondents were asked whether a health professional had ever diagnosed them with depression, anxiety, PTSD, and/or gender identity disorder (GID)/ gender dysphoria (GD). Response options were “1 = No, I don’t have this, 2 = Not sure if I have this, 3 = Yes, a health professional diagnosed me with this, 4 = Yes, I think I have this. Those indicating they had been diagnosed by a health professional were compared to all other categories (yes, no). Respondents were also asked about any mental health treatment utilization (e.g., individual psychotherapy, psychotropic medications) in the last 12 months (yes, no).

Substance use: Current smoking status was asked (“Currently, how often do you smoke cigarettes?” 0 = Not at all, 2 = Some days, 3 = Every day) and dichotomized as yes some days or every day vs. no not at all). Participants completed the AUDIT-C assessing for current alcohol use, with hazardous drinking indicated by AUDIT-C score 4+ (yes, no).[48] Past 12-month illicit drug use was assessed using a check all that apply list. Two variables were operationalized: any illicit drug use monthly or more frequently in the last 12 months, and two or more illicit drugs used monthly or more frequently in the last 12 months. Participants were asked whether they had ever in their lifetime received inpatient and/or outpatient substance abuse treatment (yes, no).

### Statistical analysis

SAS^®^ version 9.4 statistical software was used for all data analyses. Univariate descriptive statistics were obtained for all variables of interest. Distributions of individual items were assessed, including missingness. Because missingness violated assumptions required for valid statistical inferences using listwise deletion, the data were multiply imputed.[49] A fully conditional specification (FCS) imputation method was used.[50, 51] Five imputations with accompanying appropriate diagnostics were conducted, including numerical summaries that compared the observed and imputed datasets to identify any problems with imputed variables. All subsequent statistical analyses were conducted using imputed data with appropriate adjustments.

First, we examined sociodemographic characteristics associated with having a non-binary vs. binary gender identity. Non-binary vs. binary status was regressed on each demographic variable to estimate crude unadjusted odds ratios (OR) and 95% confidence intervals (95% CI). Then a single multivariable model was fit with all sociodemographic variables simultaneously with non-binary (yes, no) as the outcome. Next, analyses compared non-binary to binary transgender respondents across gender affirmation, healthcare utilization behaviors and experiences, social history, violence victimization, and social support outcomes, and mental health and substance use. For bivariate analyses, crude unadjusted regression models were estimated with non-binary vs. binary gender as a statistical predictor of health and social outcomes (logistic regression for binary variables, linear regression for continuous outcomes). Logistic regression models estimated ORs and 95% CI for binary outcomes, and linear regression models calculated beta (β) with corresponding 95% confidence limits (95% CL) for continuous outcomes. A Poisson distribution was considered for several variables (e.g., bullying frequency in childhood, number of close friends); however, model diagnostics favored a Gaussian distribution.

Multivariable regression models were then fit with non-binary vs. binary as the main statistical predictor of health and social outcomes, adjusted for the following control variables: age (continuous), gender identity (trans masculine, trans feminine spectrum), race/ethnicity (white, Black, Hispanic/ Latinx, Other, Mixed; ref = white), sexual minority (yes, no), low education (high school diploma, college or above), low income (<$35K, $35K or above), health insurance (public, private, other; ref = public). Survey mode (online, in-person) was treated as a design covariate and modeled as a fixed effect in bivariate and multivariable analyses of health and social outcomes. Adjusted OR (aOR) and 95% CI were estimated for binary outcomes and adjusted beta (aβ) and 95% CL estimates for continuous outcomes. Variables are presented in the tables for non-binary and binary respondents, as well as for the entire sample, followed by comparisons of non-binary vs. binary respondents with bivariate and multivariable parameter estimates.

## Results

### Sample characteristics

The mean age of the sample was 32.6 (standard deviation = 12.8); 63% were trans masculine; 79.4% were white non-Hispanic/Latinx, 87.8% were sexual minorities (gay, lesbian, bisexual, queer). The sample was highly educated with 85.6% college or higher education; 54.3% were higher income earning $35K annually or above. The majority (65.5%) had private health insurance, and 79.6% were recruited online. Overall, 40.9% of respondents were non-binary.

### Demographic characteristics (Table 1)

**Table 1:** Comparing the demographic characteristics of non-binary and binary transgender adults (N = 452).

	Non-Binary *n* = 185, 40.9%		Binary *n* = 267, 59.1%		Crude Models		Multivariable Models		Total Sample N = 452, 100.0%	
	Mean (SD)		Mean (SD)		β (95% CL)	p-value	aβ (95% CL)	p-value	Mean (SD)	
Age in Years (18–75)	28.95 (9.97)		35.13 (13.83)		0.96 (0.95, 0.97)	**<0.0001**	0.97 (0.96, 0.98)	**<0.0001**	32.60 (12.76)	
	N	%	n	%	OR (95% CI)	p-value	aOR (95% CI)	p-value	N	%
Gender Identity										
Trans Feminine	42	22.7	125	46.8	Ref		Ref		167	36.9
Trans Masculine	143	77.3	142	53.2	2.80 (2.31, 3.38)	**<0.0001**	1.78 (1.43, 2.21)	**<0.0001**	285	63.1
Race/Ethnicity										
White	151	81.6	208	77.9	Ref		Ref		359	79.4
Black	2	1.1	11	4.1	0.31 (0.16, 0.62)	**0.0009**	0.40 (0.19, 0.82)	**0.013**	13	2.9
Hispanic/Latinx	16	8.7	27	10.1	1.11 (0.81, 1.52)	0.523	0.98 (0.70, 1.37)	0.891	43	9.5
Other	4	2.1	9	3.4	0.69 (0.40, 1.19)	0.179	0.84 (0.47, 1.53)	0.578	13	2.9
Mixed	12	6.5	12	4.5	1.45 (1.00, 2.11)	**0.052**	1.17 (0.78, 1.77)	0.451	24	5.3
Sexual Orientation										
Not Sexual Minority	3	1.6	52	19.5	Ref		Ref		55	12.2
Sexual Minority	182	98.4	215	80.5	13.94 (8.22, 23.66)	**<0.0001**	11.95 (6.98, 20.45)	**<0.0001**	397	87.8
Education										
College or Higher	168	90.8	219	82.0	Ref		Ref		387	85.6
High School or Below	17	9.2	48	18.0	0.47 (0.35, 0.64)	**<0.0001**	0.66 (0.49, 0.90)	**0.008**	65	14.4
Current Student	69	37.3	56	21.0	2.10 (1.74, 2.54)	**<0.0001**	1.31 (1.04, 1.65)	**0.022**	125	27.6
Income										
High	102	55.0	144	53.9	Ref		Ref		246	54.3
Low	83	45.0	123	46.1	1.06 (0.89, 1.26)	0.510	1.07 (0.88, 1.31)	0.502	206	45.7
Health Insurance										
Public	34	18.6	102	38.1	Ref		Ref		136	30.1
Private	140	75.5	156	58.5	2.39 (1.94, 2.93)	**<0.0001**	1.59 (1.24, 2.04)	**0.0003**	296	65.5
Other	11	5.9	9	3.4	3.25 (2.10, 5.01)	**<0.0001**	2.72 (1.70, 4.37)	**<0.0001**	20	4.4
Survey Mode										
Online	172	93.0	188	70.4	Ref		Ref		360	79.6
In-Person	13	7.0	79	29.6	0.41 (0.30, 0.54)	**<0.0001**	0.75 (0.53, 1.05)	0.096	92	20.4

β = Beta Coefficient. aβ = Adjusted Beta Coefficient. 95% CL = 95% Confidence Limit. OR = Odds Ratio. aOR = Adjusted Odds Ratio. 95% CI = 95% Confidence Interval. Crude Models include survey mode as a design covariate. Multivariable models are adjusted for age, gender identity, race/ethnicity, sexual orientation, education, income, health insurance, and survey mode. Bolded text indicates statistical significance at the alpha 0.05-level.

Compared to binary respondents, non-binary participants were significantly younger in age (aβ = 0.97; 95% CL = 0.96, 0.98), a higher proportion were trans masculine vs. trans feminine (aOR = 1.78; 95% CI = 1.43, 2.21) and identified as sexual minority vs. not (aOR = 11.95; 95% CI = 6.98, 20.45), a lower proportion were Black (aOR = 0.40; 95% CI = 0.19, 0.82) vs. white, had lower educational attainment vs. higher (aOR = 0.66; 95% CI = 0.49, 0.90), a higher proportion were current students (aOR = 1.31; 95% CI = 1.04, 1.65) and a lower proportion had private (aOR = 1.59; 95% CI = 1.24, 2.04) or “other” health insurance (aOR = 2.72; 95% CI = 1.70, 4.37) vs. public (see Table 1).

### Gender affirmation (Table 2)

**Table 2:** Comparing gender affirmation characteristics of non-binary and binary transgender adults (N = 452).

	Non-Binary n = 185, 40.9%		Binary n = 267, 59.1%		Crude Models		Multivariable Models		Total Sample N = 452, 100.0%	
	Mean (SD)		Mean (SD)		β (95% CL)	p-value	aβ (95% CL)	p-value	Mean (SD)	
Age of Transgender Realization (0–54)	16.43 (8.33)		12.32 (8.59)		4.25 (3.53, 4.97)	**<0.0001**	3.64 (2.89, 4.41)	**<0.0001**	14.0 (8.72)	
	n	%	n	%	OR (95% CI)	p-value	aOR (95% CI)	p-value	N	%
Social Gender Affirmation (Live Full-Time)	125	67.4	219	81.9	0.47 (0.39, 0.57)	**<0.0001**	0.37 (0.30, 0.47)	**<0.0001**	344	76.1
Legal Name Change	29	15.7	153	59.5	0.13 (0.11, 0.16)	**<0.0001**	0.13 (0.10, 0.16)	**<0.0001**	182	40.3
Current and Future Medical Gender Affirmation										
Yes, Current	50	27.0	199	74.5	Ref		Ref		249	54.9
No, But I Plan to	40	21.6	45	16.9	3.72 (2.93, 4.72)	**<0.0001**	3.44 (2.65, 4.45)	**<0.0001**	85	18.8
No, I Don’t Plan to	57	30.8	6	2.2	38.66 (25.80, 57.92)	**<0.0001**	56.65 (34.51, 92.99)	**<0.0001**	63	13.9
Don’t Know	24	13.0	7	2.6	15.64 (10.35, 23.64)	**<0.0001**	28.38 (16.93, 47.58)	**<0.0001**	31	6.9
Missing	14	7.6	10	3.7	6.60 (4.46, 9.78)	**<0.0001**	6.37 (4.12, 9.85)	**<0.0001**	24	5.5
Unable to Access Transition Related Care in Last 12 Mo	30	16.2	59	22.1	0.65 (0.52, 0.81)	**0.0001**	0.51 (0.41, 0.65)	**<0.0001**	89	19.7
Medical Interventions and Body Modification Procedures										
Hormones	45	91.8	196	98.5	0.06 (0.02, 0.18)	**<0.0001**	0.02 (0.004, 0.13)	**<0.0001**	241	97.2
Non RX Hormones	9	4.9	36	13.5	0.34 (0.24, 0.47)	**<0.0001**	0.38 (0.26, 0.55)	**<0.0001**	45	10.7
Chest	16	33.3	76	38.2	0.74 (0.55, 0.99)	**0.047**	0.56 (0.39, 0.79)	**0.0009**	92	37.1
Abdominal	3	6.1	24	12.1	0.49 (0.25, 0.95)	**0.006**	0.38 (0.20, 0.71)	**0.003**	27	10.9
Genital	2	4.1	21	10.7	0.34 (0.17, 0.66)	**0.002**	0.73 (0.36, 1.51)	0.402	23	9.3
Other Procedure	4	8.3	15	7.6	1.04 (0.62, 1.75)	0.875	3.04 (1.62, 5.70)	**0.0005**	19	7.7
Silicone	1	0.5	5	1.9	0.38 (0.14, 1.00)	0.051	0.90 (0.28, 2.85)	0.854	6	1.3
Visual Nonconformity										
Low	70	37.8	160	59.8	Ref		Ref		230	50.9
Moderate	65	35.2	68	25.6	2.07 (1.70, 2.52)	**<0.0001**	1.84 (1.48, 2.28)	**<0.0001**	133	29.4
High	50	27.0	39	14.6	3.04 (2.42, 3.83)	**<0.0001**	2.84 (2.21, 3.65)	**<0.0001**	89	19.7

β = Beta Coefficient. aβ = Adjusted Beta Coefficient. 95% CL = 95% Confidence Limit. OR = Odds Ratio. aOR = Adjusted Odds Ratio. 95% CI = 95% Confidence Interval. Crude Models include survey mode as a design covariate. Multivariable models are adjusted for age, gender identity, race/ethnicity, sexual orientation, education, income, health insurance, and survey mode. Bolded text indicates statistical significance at the alpha 0.05-level. The denominator for medical interventions and body modification procedures is the number who report current medical gender affirmation (n = 50 non-binary, n = 199 binary, n = 249 total).

Compared to binary respondents, non-binary respondents recognized they were transgender and/or gender nonconforming at older ages (aβ = 3.64; 95% CL = 2.89, 4.41; Table 2). A significantly lower proportion had socially affirmed their gender (i.e., lived full-time: aOR = 0.37; 95% CI = 0.30, 0.47) and legally changed their name (aOR = 0.13; 95% CI = 0.10, 0.16). The distribution of current and future medical gender affirmation in non-binary participants was 27.0% currently affirmed their gender (referent group), 21.6% had plans to, 30.8% did not plan to, and 13.0% did not know yet (each of these proportions was significantly higher relative to currently having medically affirmed vs. binary participants). Among participants who had currently medically affirmed their gender, a lower proportion of non-binary vs. binary respondents accessed hormones in general, and non-prescription hormones specifically, as well as chest surgery, abdominal surgery, and genital surgery. A lower proportion of non-binary vs. binary respondents reported having been unable to access transition-related care in the last 12 months (aOR = 0.51; 95% CI = 0.41, 0.65). Non-binary participants had higher levels of gender nonconformity (moderate vs. low: aOR = 1.84; 95% CI = 1.48, 2.28; and high vs. low: aOR = 2.84; 95% CI = 2.21, 3.65).

### Healthcare utilization behaviors and experiences (Table 3)

**Table 3:** Healthcare utilization behaviors and experiences of non-binary and binary transgender adults (N = 452).

	Non-Binary n = 185, 40.9%		Binary n = 267, 59.1%		Crude Models		Multivariable Models		Total Sample N = 452, 100.0%	
	n	%	n	%	OR (95% CI)	p-value	aOR (95% CI)	p-value	N	%
**Last Annual Wellness Visit**										
Within 1 Year	100	57.8	213	81.9	Ref		Ref		313	72.3
Within 2 Years	44	25.4	30	11.5	2.96 (2.34, 3.74)	**<0.0001**	2.58 (2.00, 3.35)	**<0.0001**	54	17.1
Within 5 Years	19	11.0	10	3.9	3.82 (2.67, 5.47)	**<0.0001**	3.08 (2.09, 4.54)	**<0.0001**	29	6.7
5 Years or Longer	10	5.8	7	2.7	5.16 (3.04, 8.78)	**<0.0001**	7.14 (3.89, 13.08)	**<0.0001**	17	3.9
Missing n = 39										
**Healthcare Experiences, Last 12 Months**										
Had to Teach Doctor to Obtain Appropriate Medical Care	46	24.9	87	32.6	0.66 (0.54, 0.79)	**<0.0001**	0.61 (0.49, 0.75)	**<0.0001**	133	29.4
Postponed Care Resulting in Emergency Room or Urgent Care Medical Visit	20	10.8	28	10.5	0.98 (0.75, 1.29)	0.886	1.01 (0.74, 1.36)	0.974	48	10.6
Postponed Medical Care When Sick or Injured	36	19.5	51	19.1	0.96 (0.77, 1.19)	0.699	0.79 (0.62, 1.00)	0.052	87	19.2
Postponed Routine Preventive Care	49	26.5	56	20.9	1.24 (1.02, 1.52)	**0.032**	0.87 (0.70, 1.09)	0.219	105	23.2
Refused Treatment	10	5.4	14	5.2	1.00 (0.69, 1.46)	0.997	0.92 (0.61, 1.39)	0.691	24	5.3
Experienced Discrimination in a Healthcare Setting	48	26.0	68	25.2	1.03 (0.85, 1.25)	0.765	1.10 (0.89, 1.37)	0.369	116	25.7

OR = Odds Ratio. aOR = Adjusted Odds Ratio. 95% CI = 95% Confidence Interval. Crude Models include survey mode as a design covariate. Multivariable models are adjusted for age, gender identity, race/ethnicity, sexual orientation, education, income, health insurance, and survey mode. Bolded text indicates statistical significance at the alpha 0.05-level.

The proportion of non-binary respondents reporting longer time since last seeing a doctor for an annual wellness visit was significantly higher than for binary people and comparing within the last 12 months to two years (aOR = 2.58; 95% CI = 2.00, 3.35), five years (aOR = 3.08; 95% CI = 2.09, 4.54), and five years or longer (aOR = 7.14; 95% CI = 3.89, 13.08) (see Table 3). Relative to binary respondents, a lower proportion of non-binary respondents reported having to teach their doctor about transgender people to get appropriate care (aOR = 0.61; 95% CI = 0.49, 0.75).

### Social history, violence victimization, and social support (Table 4)

**Table 4:** Comparing social health history, violence victimization, and social support for non-binary and binary transgender adults (N = 452).

	Non-Binary n = 185, 40.9%		Binary n = 267, 59.1%		Crude Models		Multivariable Models		Total Sample N = 452, 100.0%	
	n	%	n	%	OR (95% CI)	p-value	aOR (95% CI)	p-value	N	%
**Social History**										
Sexually Active, Last 12 Months	150	81.1	231	86.5	0.66 (0.52, 0.83)	**0.0004**	0.73 (0.56, 0.94)	**0.014**	381	84.3
Currently Partnered	73	39.5	73	27.3	1.67 (1.39, 2.00)	**<0.0001**	1.07 (0.88, 1.30)	0.509	146	32.3
Biological and/or Adopted Children	21	11.4	47	17.6	0.58 (0.46, 0.75)	**<0.0001**	1.32 (0.95, 1.83)	0.104	68	15.0
Homeless/ Unstably Housed Ever	46	24.9	60	22.5	1.20 (0.98, 1.46)	0.080	1.34 (1.07, 1.67)	**0.010**	106	23.5
Sex Work / Transactional Sex Ever	3	1.6	11	4.1	0.54 (0.30, 0.99)	**0.045**	1.06 (0.53, 2.13)	0.868	14	3.1
Military Service Ever	4	2.2	20	7.5	0.27 (0.17, 0.44)	**<0.0001**	0.80 (0.45, 1.41)	0.432	24	5.3
**Violence Victimization**										
Intimate Partner Violence Ever	56	30.3	94	35.2	0.81 (0.68, 0.97)	**0.022**	0.80 (0.65, 0.97)	**0.023**	150	33.2
Childhood Abuse Age < 15	81	43.8	125	46.8	0.90 (0.76, 1.07)	0.226	1.10 (0.91, 1.34)	0.323	206	45.7
Physical and Sexual Abuse Age < 15										
No Abuse	104	56.2	142	53.1	Ref		Ref		246	54.4
Both Physical and Sexual Abuse	35	18.9	58	21.7	0.86 (0.69, 1.08)	0.186	1.15 (0.89, 1.49)	0.272	93	20.6
Yes, Physical Abuse Only	21	11.4	39	14.6	0.75 (0.57, 0.97)	**0.029**	0.90 (0.67, 1.20)	0.453	60	13.3
Yes, Sexual Abuse Only	25	13.5	28	10.6	1.18 (0.91, 1.55)	0.215	0.31 (0.98, 1.76)	0.074	53	11.8
	Mean (SD)		Mean (SD)		β (95% CL)	p-value	aβ (95% CL)	p-value	Mean (SD)	
Bullying Frequency in Childhood (0–4)	2.62 (1.07)		2.62 (1.10)		-0.02 (-0.12, 0.08)	0.732	-0.09 (-0.20, 0.01)	0.090	2.62 (1.09)	
**Social Support**										
Family Support (0–3)	1.60 (0.95)		1.80 (1.07)		-0.20 (-0.29, -0.11)	**<0.0001**	-0.26 (-0.36, -0.16)	**<0.0001**	1.72 (1.02)	
Number Close Friends	4.46 (2.82)		4.27 (3.01)		0.18 (-0.07, 0.43)	0.150	-0.14 (-0.40, 0.12)	0.302	4.35 (2.93)	
Number Close Transgender Friends	1.86 (1.80)		1.96 (2.72)		-0.08 (-0.30, 0.13)	0.427	-0.12 (-0.35, 0.11)	0.295	1.92 (2.39)	

β = Beta Coefficient. aβ = Adjusted Beta Coefficient. 95% CL = 95% Confidence Limit. OR = Odds Ratio. aOR = Adjusted Odds Ratio. 95% CI = 95% Confidence Interval. Crude Models include survey mode as a design covariate. Multivariable models are adjusted for age, gender identity, race/ethnicity, sexual orientation, education, income, health insurance, and survey mode. Bolded text indicates statistical significance at the alpha 0.05-level.

Non-binary respondents had decreased odds of being sexually active (aOR = 0.73; 95% CI = 0.56, 0.94) and increased odds of having ever been homeless (aOR = 1.34; 95% CI = 1.07, 1.67) relative to binary respondents (Table 4). No differences were found in relationship status, parental status, and previously or currently being in the military. A significantly lower proportion of non-binary respondents reported lifetime IPV vs. binary respondents (aOR = 0.80; 95% CI = 0.65, 0.97); no other statistically significant differences in violence victimization were observed. Non-binary respondents had lower levels of family support for their gender than binary respondents (aβ = -0.26; 95% CL = -0.36, -0.16); no significant differences were found in the number of friends or transgender friends.

### Mental health and substance use (Table 5)

**Table 5:** Comparing mental health and substance use for non-binary and binary transgender adults (N = 452).

	Non-Binary n = 185, 40.9%		Binary n = 267, 59.1%		Crude Models		Multivariable Models		Total Sample N = 452, 100.0%	
	Mean (SD)		Mean (SD)		β (95% CL)	p-value	aβ (95% CL)	p-value	Mean (SD)	
**Mental Health**										
Internalized Transphobia (Wish Not Transgender) (0–4)	2.41 (1.11)		2.74 (1.32)		-0.35 (-0.46, -0.23)	**<0.0001**	-0.26 (-0.38, -0.14)	**<0.0001**	2.61 (1.25)	
	n	%	n	%	OR (95% CI)	p-value	aOR (95% CI)	p-value	N	%
Self-Harm, Lifetime	93	50.2	152	46.8	1.11 (0.93, 1.31)	0.245	0.76 (0.63, 0.92)	**0.006**	218	48.2
Suicide Attempt, Lifetime	59	31.9	90	33.7	0.97 (0.81, 1.16)	0.732	1.20 (0.98, 1.46)	0.083	149	32.9
Depression CES-D-10, Past 7 Days	48	25.9	59	22.1	1.20 (0.99, 1.47)	0.068	1.42 (1.14, 1.77)	**0.002**	107	23.7
Depression Diagnosis, Lifetime	85	46.0	128	47.9	0.93 (0.78, 1.10)	0.376	0.83 (0.69, 0.99)	**0.048**	213	47.1
Anxiety Diagnosis, Lifetime	70	37.8	120	44.9	0.75 (0.63, 0.90)	**0.001**	0.56 (0.47, 0.68)	**<0.0001**	190	42.0
PTSD Diagnosis, Lifetime	39	21.1	59	22.1	0.96 (0.78, 1.18)	0.706	0.98 (0.78, 1.23)	0.877	98	21.7
GID/ GD Diagnosis, Lifetime	22	11.9	34	12.7	0.93 (0.72, 1.20)	0.563	0.72 (0.55, 0.96)	**0.024**	56	12.4
Any Mental Health Treatment, Last 12 Months	76	41.3	129	48.3	0.79 (0.67, 0.94)	**0.007**	0.75 (0.62, 0.91)	**0.003**	205	45.4
**Substance Use**										
Current Smoker	73	39.5	94	35.2	1.25 (1.05, 1.48)	**0.014**	1.10 (0.91, 1.34)	0.318	167	36.9
AUDIT-C Hazardous Drinking	72	44.7	92	38.3	1.25 (1.04, 1.50)	**0.016**	1.24 (1.01, 1.52)	**0.038**	164	36.3
Any Illicit Drug, Last 12 Mo	83	44.9	111	41.6	1.19 (1.00, 1.41)	0.051	1.13 (0.94, 1.37)	0.198	194	42.9
Two or More Illicit Drugs, Last 12 Mo	33	17.8	43	16.1	1.18 (0.94, 1.47)	0.158	1.22 (0.95, 1.56)	0.122	76	16.8
Substance Abuse Treatment, Lifetime	14	7.6	31	11.6	0.66 (0.50, 0.91)	**0.010**	1.05 (0.75, 1.48)	0.768	45	10.0

OR = Odds Ratio. aOR = Adjusted Odds Ratio. 95% CI = 95% Confidence Interval. Crude Models include survey mode as a design covariate. Multivariable models are adjusted for age, gender identity, race/ethnicity, sexual orientation, education, income, health insurance, and survey mode. Bolded text indicates statistical significance at the alpha 0.05-level.

Compared to binary respondents, non-binary respondents had lower levels of internalized transphobia (aβ = -0.26; 95% CL = -0.38, -0.14; Table 5). Non-binary respondents also had decreased odds of lifetime self-harm (aOR = 0.76; 95% CI = 0.63, 0.92), diagnosis of anxiety (aOR = 0.56; 95% CI = 0.47, 0.68), diagnosis of depression (aOR = 0.83; 95% CI = 0.69, 0.99), and diagnosis of GID/ GD (aOR = 0.72; 95% CI = 0.55, 0.96) relative to binary respondents. Despite having increased odds of clinically significant depressive distress in the last 7 days (aOR = 1.42; 95% CI = 1.14, 1.77) relative to binary respondents, non-binary respondents had decreased odds of receiving any mental health treatment in the last 12 months (aOR = 0.75; 95% CI = 0.62, 0.91). For substance use, non-binary respondents had an increased odds of a positive AUDIT screen for hazardous alcohol use (aOR = 1.24; 95% CL = 1.01, 1.52) relative to binary respondents.

## Discussion

In this community-recruited sample of transgender and gender nonconforming adult respondents in Massachusetts, 40.9% identified their gender identity as non-binary, a proportion of the sample comparable to other research with transgender people in the U.S.[11] Consistent with hypotheses, described in detail below, differences were found by participant demographics, gender affirmation status, mental health, substance use, social history, violence/victimization, and social support for non-binary and binary respondents. Findings suggest that gender diversity in transgender people, specifically, whether a person has a self-identified, non-binary or binary gender identity, is an important consideration for transgender health research and clinical care. Findings add to the nascent health research literature about the social and health status of non-binary people.[10] Gender identity measures that include binary and non-binary response options are recommended to inform future research and clinical care with transgender populations.

In the statewide sample of transgender adults, a number of socio-demographic differences were found between non-binary and binary participants. As expected, on average, non-binary participants were younger in age than binary respondents, consistent with other research.[11, 14] Gender differences also emerged, such that a higher proportion of non-binary vs. binary adults were trans masculine rather than trans feminine (e.g., assigned a female sex at birth compared to male sex)–a finding which aligns with previous research.[14, 52] This difference may, at least in part, reflect how gender non-conformity is more socially acceptable for individuals assigned a female sex at birth than those with a recorded male birth sex.[53, 54] The overwhelming majority of non-binary respondents (98.4%) self-identified as having a sexual minority sexual orientation, which is also consistent with findings from other research.[11, 32] This substantial overlap between non-binary gender and sexual minority status is intriguing and supports the conceptualization that “non-traditional” gender identities (i.e., outside the gender binary) and sexual orientation are distinct yet interrelated constructs. More research is needed to understand the terms non-binary people use to describe and identify their sexual orientation identity, as well as the processes of gender identity and sexual orientation identity development.

Genderqueer and other non-binary identity terms have been critiqued for disproportionately representing the experiences of white non-Hispanic/Latinx people and more highly educated and better-resourced subgroups of transgender people.[13] Consistent with these critiques, we found that a lower proportion of non-binary participants were people of color and a higher proportion of participants were more highly educated as well as current students. Additionally, while no significant differences emerged by gender identity in terms of income level, non-binary individuals did have a higher frequency of private and “other” health insurance compared to binary respondents. It is possible that white transgender people and those with higher SES may be afforded the privilege to live outside the gender binary and thus be more likely to utilize non-binary identity terms. Gender identity terms and gender expression may also differ by race and class,[45] and thus is possible that a higher proportion of low SES transgender individuals and people of color actually live outside the gender binary but do not use non-binary identity terms such as genderqueer or gender fluid. Mixed methods research is needed to further explore socio-demographic differences according to gender identity as well as expression.

In the present sample, hypothesized differences were also found with respect to gender affirmation. On average, non-binary participants realized they were transgender at an older mean age than binary respondents, potentially suggesting a different developmental trajectory and age pattern of gender identity self-recognition. This finding, while aligning with previous research,[52] is particularly striking given that non-binary respondents were younger, on average, than binary respondents. Further, a significantly lower proportion of non-binary vs. binary respondents had socially, legally, and medically affirmed their gender, which is consistent with findings from other research.[11, 52] Still, more than one-fourth (27.0%) of non-binary participants had medically affirmed their gender, with 21.6% planning to pursue medical gender affirmation and 30.8% not planning to pursue it. More than 1 in 10 (13.0%) non-binary respondents were not sure whether or not they planned to access medical gender affirmation in the future. Among those who had accessed medical gender affirmation, a significantly lower proportion of non-binary vs. binary respondents reported non-prescription hormone use (4.9% vs. 13.5%). The heightened use of non-prescription hormone use among binary participants may be reflected by the fact that 22.1% of binary participants reported being unable to access transition-related care in the last 12 months, a proportion significantly higher than for non-binary respondents (16.2%). These findings are consistent with prior research suggesting that transgender individuals may use non-prescription hormones when unable to access care through a healthcare provider.[55] The heterogeneity of medical affirmation desires and utilization underscores the importance of ensuring access to care for all transgender individuals, as well as individualized and patient-centered care by gender identity subgroups. Developing clinical tools that will facilitate delivering patient-centered care is important to meeting the gender affirmation needs of diverse transgender adults, including non-binary individuals.[56]

As hypothesized, differences were also observed in some areas of healthcare utilization according to gender identity. Specifically, non-binary participants had increased odds of not having an annual wellness visit for primary care within the last two or more years, indicating lower levels of routine healthcare engagement. Contrary to hypotheses, a lower proportion of non-binary respondents reported having to teach their doctor or provider about transgender people in order to get appropriate care (24.9% vs. 32.6%), relative to binary participants. The USTS found that 84% of binary individuals reported discussing their gender identity with their healthcare provider, relative to 52% of non-binary respondents.[11] Given the USTS findings and the fact that non-binary participants in the present study were less likely to access medical gender affirmation than binary participants, it may be that non-binary individuals do not feel the need to disclose their identity to providers, therefore limiting the need to educate their provider about transgender or non-binary people. However, non-binary individuals were significantly more likely to be visually gender nonconforming than binary participants, thus it is likely that providers observed their patients’ visual nonconformity, which may help to explain the lack of significant differences between binary and non-binary participants with respect to other aspects of healthcare utilization. For example, the rates of postponing healthcare and experiencing discrimination in the past 12 months were nearly equal for non-binary and binary participants (e.g., experiencing mistreatment in healthcare 26.0% vs. 25.2%; being refused healthcare 5.4% vs. 5.2%; and postponing care when sick or injured 19.5% vs. 19.1%). These findings differ slightly from the national research findings showing a higher proportion of binary participants had experienced some form of healthcare discrimination than non-binary (39% vs. 24%) and were also more likely to have avoided healthcare for fear of discrimination in the past year (27% vs. 20%).[11] Nonetheless, the findings reported here suggest that it is the experience of being transgender that places Massachusetts’ residents at risk for negative healthcare experiences, rather than their gender identity, and that binary and non-binary transgender individuals respond similarly to the threat of discrimination (e.g., healthcare avoidance). Additional qualitative research with healthcare providers and transgender patients may help to further explain differences and similarities in healthcare engagement experiences and behaviors according to gender identity.

Differences in social history and social support were not anticipated by gender identity; however, several key differences did emerge. Non-binary participants were more likely to have ever been homeless/unstably housed than binary individuals, which may be explained by the fact that non-binary participants also had lower levels of support from family regarding their gender identity than binary respondents. These findings are consistent with the USTS, which found that the prevalence of homelessness was substantially higher among respondents whose immediate family had kicked them out of the house, with nearly three-quarters (74%) of these respondents experiencing homelessness.[11] While family rejection was higher for non-binary participants in the present sample, contrary to what was anticipated,[19] no significant differences were found in the prevalence of childhood sexual abuse, bullying, lifetime physical and sexual assault, or relevant protective factors such as number of close friends. Indeed, non-binary participants did report significantly lower lifetime experiences of IPV relative to binary participants, which could be explained in part by the finding that non-binary individuals were less likely to be sexually active, though no differences were found according to current relationship status. Additional research examining health risk trajectories and protective factors across the life-course is needed to contextualize experiences of victimization and support among transgender individuals by gender identity subgroup.

Finally, mental health and substance use outcomes differed by gender identity, albeit differently than hypothesized. In terms of mental health, non-binary participants were less likely to report lifetime self-harm, which may be explained by the significantly lower prevalence of internalized transphobia experienced by non-binary participants relative to binary participants. Non-binary individuals in this sample were also less likely than binary individuals to have been diagnosed with depression and anxiety by a healthcare provider. The lower prevalence of depression and anxiety diagnoses among non-binary participants in the study likely reflects differential access to opportunities to receive a medical diagnosis as, consistent with other studies,[8, 11, 57] a significantly higher proportion of non-binary screened-in for current clinically significant depressive distress, yet were less likely to have received mental health care treatment in the last 12 months relative to binary individuals. Additionally, a higher proportion of non-binary vs. binary respondents screened positive for hazardous drinking, which may in part be due to the younger age of the non-binary respondents. However, elevated hazardous drinking may also suggest an attempt to self-medicate, particularly in light of this sub-population’s lower engagement in behavioral healthcare. Findings suggest a potential unmet need for mental/behavioral healthcare services for non-binary transgender people, particularly in the context of current depressive distress and problematic alcohol use. Efforts may be needed to increase screening for current mental/behavioral health problems in clinical care settings, including in community-based healthcare centers with large transgender patient panels.

Findings from this study should be interpreted in light of several limitations. Non-probability sampling methods were used; thus, results are likely not generalizable to other states or geographic locales. The cross-sectional nature of these data means that results are associational only; findings require replication in other studies with different study designs (e.g., longitudinal) to overcome the limitations of these data. Additionally, the current study was not able to look at multiple gender identity groupings and permutations; future research might pursue this line of work to consider intersectional identities. Due to sample size limitations, it was not possible to stratify models by gender identity spectrum (e.g., as trans masculine vs. trans feminine) in considering non-binary vs. binary health; however, all multivariable models were adjusted for gender identity spectrum. For some health indicators, the association of non-binary status and health may be moderated by gender identity spectrum. Future research is needed with larger sample sizes in order to test for effect modification by assigned sex at birth for non-binary health associations. Additionally, one of the criteria for inclusion in this study was being transgender and/or gender nonconforming. This way of operationalizing the study sample differs quite dramatically from other approaches, particularly those that use clinical diagnostic thresholds for gender dysphoria.[58] It may be that non-binary respondents were more likely to identify as gender nonconforming than transgender, and may not have selected into a study of transgender people only. Unfortunately, we did not collect data that would allow us to answer this question. Future research would benefit from an understanding of how the population definition influences non-binary people’s participation.

The present study used a two-step method for gender identity whereby participants were asked to select a single response option that best described their current gender identity, and were subsequently categorized as non-binary and binary. The two-step method is recommended to capture transgender status based on current gender identity and sex assigned at birth.[45] The method was developed to identify transgender respondents and enable between-group comparisons of transgender and cisgender for population health surveys. Findings from the current study demonstrate that the two-step method can also be applied to characterize within-group differences among transgender people, such as this study where we compared the health of non-binary and binary transgender identity groups. Formal validation testing of the two-step method for capturing non-binary vs. binary gender identities has not, to our knowledge, been conducted. However, a cognitive testing study of the two-step method did find that non-binary respondents most frequently checked the response option “do not identify as male, female, or transgender”, rather than male, female, or transgender response options.[59] Given the high proportion of non-binary individuals in this sample, and in other recent research with transgender people,[11] the use of the two-step method is recommend with response options that will allow for non-binary identities to be categorized. This might include a response option of “genderqueer” or “non-binary”, in addition to “transgender man” or “transgender woman”; or it may be “do not identify as male, female, or transgender.” Another option, if skip patterns are possible, would be to ask individuals who self-identify as transgender subsequent questions about their specific gender identity. While this method is recommended for all research assessing gender identity, it should be noted that transgender individuals might identify as multiple gender categories (i.e., as trans men and non-binary). Participants in the present study were not given the option to “check all that apply” for gender identity; however, future work with this population should consider providing respondents with two gender identity questions that include a forced (“pick the best one”) and “check all that apply” option in order to assess the range of possible identity categories endorsed by transgender populations.

Limitations notwithstanding, this study provides much-needed data about non-binary people and compares the health and social history of this group to binary-identified transgender adults, motivated by minority stress frameworks.[15–17] The growing recognition that gender diversity does not equate to gender pathology is bringing medical care for transgender people into the “mainstream.” Primary care, general internal medicine, and family practice physicians need more epidemiological knowledge and information to manage the diverse clinical presentations they see in practice with transgender patients.[12, 20] Implementing informed consent protocols for treatment will bring physicians into greater contact with transgender patients, including those who present with diverse gender identities and presentations.[60] Demographic measures of gender identity that include binary and non-binary response options are recommended to inform future research and clinical care with transgender populations.

### What is already known on this subject?

Transgender health research and epidemiology has yet to consider whether people who identify as non-binary have different health profiles than those endorsing a binary gender identity. These data are urgently needed to guide public health efforts and inform clinical care responsive to the lived experiences of diverse transgender people.

### What this study adds?

Non-binary people differ on key demographic, social, and health indicators than binary-identified transgender people. Gender diversity is an important consideration for transgender health. Demographic measures of gender identity that include binary and non-binary response options are recommended to inform future public health research and clinical care.
